# The path to equitable HIV prevention

**DOI:** 10.1038/s43856-022-00224-2

**Published:** 2022-12-12

**Authors:** John Alechenu Idoko, Beatriz Grinsztejn, Nittaya Phanuphak

**Affiliations:** 1 Department of Medicine, Infectious Diseases Unit, University of Jos, Jos, Nigeria; 2 Federal University of Health Sciences, Otukpo, Nigeria; 3 Evandro Chagas National Institute of Infectious Diseases-FIOCRUZ, Rio de Janeiro, Brazil; 4 Institute of HIV Research and Innovation, Bangkok, Thailand

## Abstract

HIV remains a major global health issue, with the burden of the epidemic disproportionately falling on low- and middle-income countries. Progress in HIV prevention, most notably pre-exposure prophylaxis (PrEP), has been slow to reach those most in need.

In this Viewpoint, three HIV researchers describe some of the barriers in access to HIV prevention affecting their regions, from Latin America to sub-Saharan Africa and Southeast Asia. The authors outline strategies to help achieve more equitable HIV prevention and mitigate HIV transmission in low- and middle-income countries.

## John Alechenu Idoko

**Figure:**
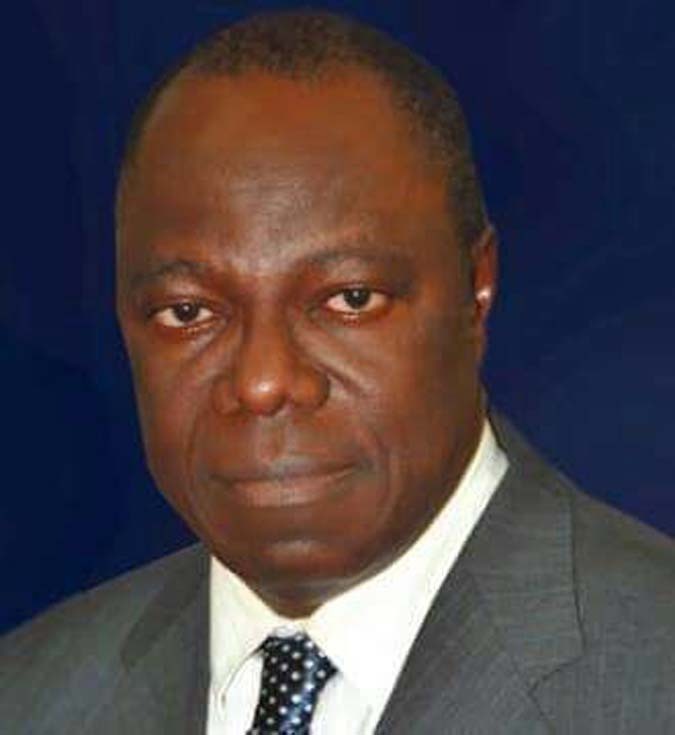
© the Author

I am a physician trained in internal medicine, infectious diseases, and immunology of infectious diseases with over 25 years of work, research and leadership experience in HIV/AIDS. I was Principal Investigator of the Harvard University/AIDS Prevention Initiative Nigeria (APIN) program, one of the largest United States President Emergency Plan for AIDS Relief (PEPFAR)-funded HIV clinics in Nigeria at the University of Jos, Nigeria, with over 15,000 patients receiving life-saving anti-retroviral therapy. I was also the Principal Investigator of the Nigerian Pre-Exposure Prophylaxis Research Program supported by Georgetown University and Bill & Melinda Gates Foundation.

Enormous progress has been made in the HIV response since the beginning of the HIV pandemic. Fewer people are dying from AIDS and new infections are declining. However, the progress made has been uneven across different parts of the world and across different populations in the same country. For example, the 2018 Nigerian HIV/AIDS Indicator and Impact Survey (NAIIS) showed that despite the significant decline in the HIV prevalence in the general population, young women aged between 20 and 24 years had a HIV prevalence four times that of their male peers^1^. In low- and middle-income countries (LMICs), coverage of HIV services has been inadequate and has not reached many people at risk. Out of the 37 million people living with HIV globally, 17 million are unaware of their HIV status and most of these live in LMICs^2^. In countries where same sex relationships, sex work and drug use are criminalized, levels of HIV knowledge and testing are low, and stigma is a major issue. Gender-based violence robs women of their human rights, including their rights to education, health and economic opportunities. It is also associated with a high risk of contracting HIV.

Over the last 2 and a half years, the COVID-19 pandemic has had a devastating effect on the HIV response in LMICs, exposing and worsening the inequalities and weaknesses in health systems. HIV prevention efforts were compounded by the shutdown of services during the COVID-19 pandemic lockdowns, which exacerbated HIV transmission. National data from Uganda observed increased sexual violence among girls with increased HIV exposure during the lockdowns^3^.

Promoting equitable access to HIV prevention in LMICs will require strengthening combination prevention with new tools including the use of PrEP^4^, which can now be implemented with long-acting injectable antiretroviral therapy (ART), which does not require daily oral dosing; expanding voluntary medical male circumcision, which has been shown in three randomized trials in South Africa, Kenya and Uganda to reduce new HIV infections by 60%;^5^ and promoting the consistent use of male and female condoms. Other innovative tools include the use of topical virucidal agents and, eventually, it is hoped that HIV vaccines will prove a powerful tool for HIV prevention. Implementation of combination prevention needs to be supported by the systematic collection of data to observe and address inequalities and understand the drivers and geographical patterns of the epidemic in each LMIC.

Promoting equity in HIV prevention in LMICs will require testing millions who do not know their HIV status, commencing and retaining them on ART. This will require strong political will, expanding and strengthening human resources, logistics, strengthening of health systems, community mobilization and providing adequate resources. Innovative and pragmatic ways should be explored to reach key vulnerable populations and others outside the reach of the traditional health system. With vertical transmission remaining a major problem in LMICs, the elimination of mother-to-child transmission of HIV will require a comprehensive program to provide women with services as they become sexually active and plan their families, with follow up through pregnancy, childbirth and breast-feeding.

To further strengthen HIV prevention, it is necessary to address structural factors such as improving children’s education (particularly that of girls), preventing gender-based violence and promoting community mobilization to change behavior associated with a risk of contracting HIV. Other considerations include the removal of laws, policies and practices that discriminate against and stigmatize people living with HIV, including vulnerable populations. Finally, with millions of children and adolescents living with HIV or affected by HIV globally, young people need to be mobilized to leverage social media, a preferred mode of communication, to educate each other and promote access to and uptake of HIV prevention.

## Beatriz Grinsztejn

**Figure:**
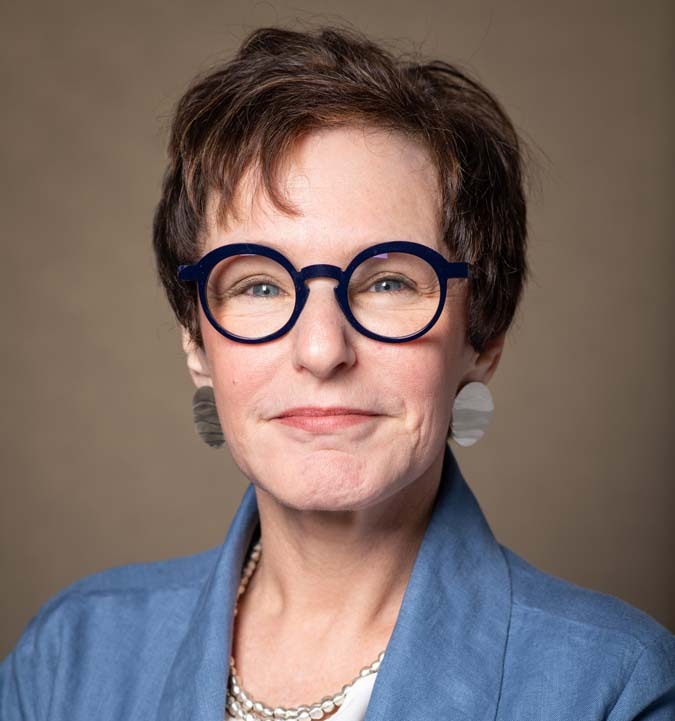
© the Author

I am an infectious diseases physician and researcher at the Evandro Chagas National Institute of Infectious Diseases-Oswaldo Cruz Foundation in Rio de Janeiro, Brazil. With more than 35 years working in the HIV field, I have dedicated my career to improving people’s lives and contributing to scientific knowledge on HIV prevention and treatment, with a major focus on vulnerable populations. As a clinical investigator, I have led and participated in seminal studies that changed the HIV prevention and care landscape globally.

In the past decade, there has been a 31% reduction in new HIV infections diagnosed annually across the world^6^. The HIV prevention landscape now provides a wider range of choices to better suit evolving needs, particularly those of people most vulnerable to infection^7^. In Latin America, the key at-risk populations include gay men and other men who have sex with men (MSM), and transgender women^8,9^. Yet while the continuum of care in Latin American countries has improved over time and sustained ART coverage has led to a decrease in AIDS-related mortality, there are worrying signs regarding access to HIV prevention methods^7^. The annual number of new HIV infections has barely changed over the past two decades, with no country in the region reaching the UNAIDS 95-95-95 targets^8^^,^ which aim for 95% of those living with HIV to know their status, 95% of those who know their status to be on treatment, and 95% of those on treatment to be virally suppressed. In 2018, substantial percentages of MSM, travestis (a term proudly adopted by many Brazilian persons who were assigned male at birth but have a gender identity within the female spectrum), and transgender women, had never been tested for HIV. Although in 2021 this proportion decreased, regular HIV testing is still not a widespread practice^10^. Despite achievements in access to ART, over one-third of individuals are still diagnosed with advanced HIV infection, and AIDS-related conditions remain the leading causes of death among people living with HIV^6^.

Despite the recommendation by the World Health Organization in 2014 of daily oral HIV PrEP for individuals at higher vulnerability^11^, its availability has been very limited in Latin America. In 2020, only 10% of PrEP users from 77 countries were in Latin America^12^, despite the stable number of new HIV infections among vulnerable populations in the region in the last 2 decades^6^. Although PrEP awareness and willingness to use in the region are high, its adoption as a public health policy has been very limited^12–14^.

The requirement for consistent adherence to oral PreP can make this prevention strategy quite challenging for those who suffer structural oppressions, reinforcing how inequities might negatively impact PrEP uptake, adherence and persistence^7,15^. The vulnerabilities faced by key populations, ranging from stigma and discrimination, physical and sexual harassment, violence, and other human rights violations undermine access to the full range of HIV prevention options. Compensating for these inequities, that lie at the root of high HIV vulnerability, low service uptake and retention, is paramount.

New prevention strategies are crucial and may directly impact on individual-level risks, necessary for the spread of disease. Nevertheless, they will be insufficient to reach the population-level epidemic dynamics if not successfully implemented. Long-acting PrEP is a potential game-changer for populations whose social vulnerabilities contribute to poorer PrEP outcome^16^. Hopefully access barriers will not hinder the benefits for those most in need. Increased awareness and tackling of regional inequities^15^ will require consolidated action from diverse stakeholders. First, policymakers will need to focus on making PrEP availability a public health policy priority in Latin America^8^. It is also necessary to use implementation science in HIV prevention research, as an opportunity to identify barriers and maximize feasibility and acceptability. While close to 90% of countries in Latin America are implementing social protection strategies or policies, advocacy efforts are required to ensure that these more intentionally benefit key populations living with HIV. Research is an investment that needs to be made, and some studies^17^ have shown that increased donor presence can reduce the global health disease burden. However, there are very few donors in the region considering levels of inequity^18^. Those that are present should utilize their diplomatic voice and resources to substantively increase program funding for HIV prevention in key populations. Lastly, while the Undetectable = Untransmittable (U = U) campaign—which aims to spread awareness that someone with an undetectable HIV viral load on HIV treatment cannot transmit HIV—is credited with beginning to change the public perception about HIV transmissibility, its application across the region remains limited^19^.

As the most unequal region in the world, Latin America suffers the consequences of its inequalities^20^. These inequalities are a major driver of the HIV epidemic, which continues to disproportionately burden vulnerable populations. Our current models of HIV prevention delivery are not responding to the needs of these populations. The gaps in HIV responses and resulting HIV infections and AIDS-related deaths lie upon inequality, and the most vulnerable populations struggling with poverty and social inequalities are the ones that suffer the most from HIV.

## Nittaya Phanuphak

**Figure:**
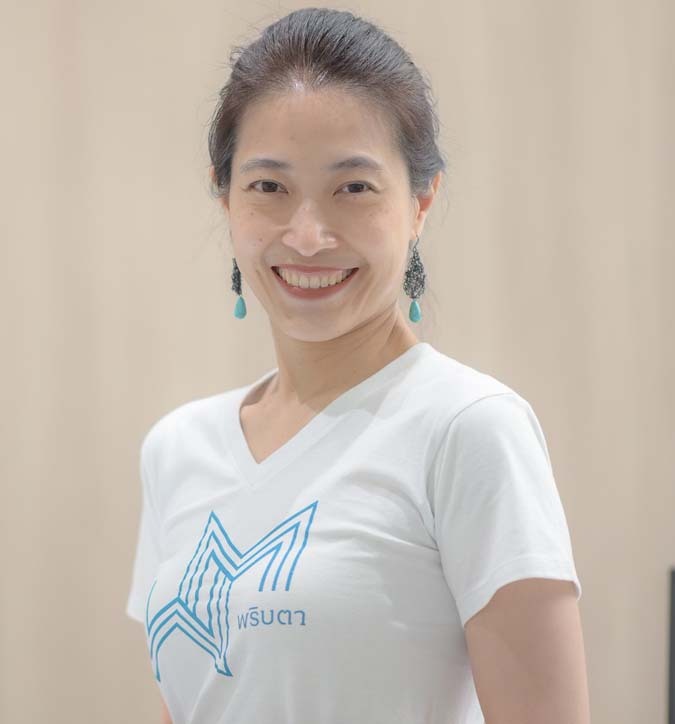
© Institute of HIV Research and Innovation

As the Executive Director of the Institute of HIV Research and Innovation (IHRI) in Bangkok, Thailand, I lead HIV prevention clinical trials and implementation research aiming at rapid translation of science into wide-scale practice, as well as evidence-based health policy changes, in Thailand and the Asia Pacific.

Rapid and large-scale implementation of evidence-based HIV prevention interventions have successfully curbed new HIV infections in a few high-income cities. An HIV status-neutral approach is used in these settings putting equal importance on treatment and prevention. People living with HIV receive immediate ART initiation aiming at achieving undetectable viral load to ensure zero risk of sexual transmission of HIV. Individuals without HIV are offered PrEP and other HIV prevention choices aimed at achieving negligible risk of HIV acquisition.

Southeast Asia has 66,200 new HIV infections each year, with the highest numbers of 17,000 in the Philippines and 28,000 in Indonesia. PrEP scale-up has varied among countries. Thailand and Vietnam have the highest number of cumulative PrEP users at 41,027 and 33,938 by March 2022. Thus far, there are three key components for a successful PrEP scale-up in the region. The first component is the availability of nationally registered generic oral PrEP products to reduce costs. The second is the formal development of national PrEP guidelines. The third is the type of PrEP service delivery models endorsed at a national level. The PrEP service delivery model which has contributed most to expand PrEP access in Thailand and Vietnam is the key population (KP)-led PrEP service where lay providers who are members of KP communities are trained and certified to provide same-day PrEP services to their community in KP-led clinics. Clients of these KP-led clinics include MSM, transgender people, sex workers, and people who use substances. They are reached through popular online social network platforms virtually and at in-person gatherings. In Thailand, KP-led PrEP served 82% of current PrEP users in 2021.

The US President’s Emergency Plan for AIDS Relief and the Global Fund to fight AIDS, Tuberculosis, and Malaria have also influenced HIV prevention efforts in many countries in Southeast Asia. Instead of granting budget to run business as usual, funding agencies should mandate the use of the fund to catalyze the implementation of evidence-based HIV prevention interventions through innovative KP-led service delivery models. This must be done with an end in mind to transition financial support and capacity building to in-country partners for sustainability.

Research in Thailand and Vietnam contributed significantly to the US FDA’s approval of long-acting cabotegravir (CAB-LA), which was proven to be superior to oral daily PrEP in preventing HIV infection. In its current form, CAB-LA needs to be injected by healthcare providers every two months, after the first two doses given one month apart. We now need to ensure timely registration of the CAB-LA product, with an affordable price in Southeast Asian countries. CAB-LA and oral PrEP must be positioned as equal options to allow users to be able to choose the approach that suits them. We also need to seriously explore the feasibility of integrating CAB-LA in the KP-led PrEP service through implementation research. Only with concerted effort made among communities, health authorities, researchers, and policy makers can we bring equitable HIV prevention to the region.
